# Synergistic combinations of paclitaxel and withaferin A against human non-small cell lung cancer cells

**DOI:** 10.18632/oncotarget.27519

**Published:** 2020-04-21

**Authors:** Al Hassan Kyakulaga, Farrukh Aqil, Radha Munagala, Ramesh C. Gupta

**Affiliations:** ^1^ Department of Pharmacology and Toxicology, University of Louisville, Louisville, KY 40202, USA; ^2^ Department of Medicine, University of Louisville, Louisville, KY 40202, USA; ^3^ James Graham Brown Cancer Center, University of Louisville, Louisville, KY 40202, USA

**Keywords:** non-small cell lung cancer, withaferin A, synergistic, combination index, paclitaxel

## Abstract

Platinum-taxane combination chemotherapy still represents the standard of care for advanced non-small cell lung cancer (NSCLC) with no targetable driver mutations. However, the efficacy of these drugs has plateaued at 10–14 months primarily due to dose-limiting toxicity, chemoresistance, and metastasis. Here, we explored the effects of withaferin A (WFA) alone and in combination with paclitaxel (PAC) on the growth, proliferation, migration, and invasion of human NSCLC cells. We show that the sensitivity of H1299 and A549 cells to concomitant treatment with PAC and WFA was greater than that of either PAC or WFA alone. Using the combination index and dose-reduction index, we demonstrated that various combinations (1:40, 1:20, 1:10) of PAC to WFA, respectively, were highly synergistic. In addition, PAC+WFA co-treatment synergistically inhibited colony formation, migration, invasion and increased the induction of apoptosis in H1299 and A549 cells. Interestingly, the synergism of PAC and WFA was not schedule-dependent but was enhanced when cells were pretreated with WFA indicating a chemo-sensitizing effect. Importantly, WFA was active against both PAC-sensitive (TS-A549) and PAC-resistant (TR-A549) cells both *in vitro* and *in vivo*. Mechanistically, WFA inhibits the proliferation of NSCLC cells via thiol oxidation. The effects of WFA were inhibited in the presence of N-acetyl cysteine and other thiol donors. Taken together, our results demonstrate the efficacy of WFA alone or alongside PAC on NSCLC cells and provide a strong rationale for further detailed testing in clinically relevant models for the development of PAC+WFA combination as an alternative therapeutic strategy for advanced NSCLC.

## INTRODUCTION

Lung cancer remains the leading cause of cancer-related deaths among both men and women in the U. S and worldwide [1, 2]. This extremely poor prognosis is explained in part by three main characteristics of lung cancer: a) distant organ metastasis at diagnosis, b) a high degree of cellular and genetic diversity, and c) rapid development of drug resistance [3, 4]. Clinically, non-small cell lung cancer (NSCLC) represents 85–90% of all the cases of lung cancer and has an overall five-year survival rate of 15% [5, 6]. The therapeutic options for NSCLC following diagnosis are dependent upon the clinical stage at diagnosis [6–8] with surgical resection being the standard of care for early-stage NSCLC [9]. However, in 60–70% of NSCLC cases, tumors are either locally advanced or extensively metastatic at diagnosis [10, 11]. As such, systemic chemotherapy is the only viable, effective and cornerstone therapeutic strategy for the treatment of advanced NSCLC [12, 13]. Indeed, decades of clinical trials have demonstrated that chemotherapy relieves disease symptoms and improves the quality of life in NSCLC patients [14].

The choice of chemotherapeutic regimen in NSCLC is mainly determined by the histological sub-type and the status of genetic driver mutations [10, 14]. Recently, landmark discoveries of targeted drugs [14, 15] and immunotherapies [16, 17] have shifted the frontline therapies towards an era of personalized medicine [5, 17, 18]. However, besides the high cost of therapy, the targeted drugs and immunotherapies benefit small and specific groups of NSCLC patients. This is true primarily because < 30% of all NSCLC patients show targetable mutations [19], while anti-PD-1 drugs are effective only in patients with tumors expressing PDL-1 on >50% of tumor cells [20]. Moreover, drug resistance during the course of treatment is a major limiting factor in targeted and immunotherapies. Therefore, despite these recent therapeutic advancements, NSCLC remains largely incurable, and the overall clinical benefit of current therapies in NSCLC is still marginal and temporary [6, 8].

Platinum-based chemotherapies are still the first-line regimens in the treatment of NSCLC cases with no targetable genetic mutations [21, 22]. Patients are administered with four-six cycles [22] of platinum-based chemotherapy, which normally consists of a platinum compound such as cisplatin (cis-Pt) or carboplatin administered alongside a third-generation chemotherapeutic agent [10, 12, 21, 22]. In the clinic, the taxane-platinum combinations are the standard of care treatments of advanced NSCLC [14, 23]. Taxanes as a class of anticancer agents is a large group of compounds that target microtubule function during cell division [24]. Paclitaxel (PAC) the most prominent taxane was first isolated from extracts of the bark of *Taxus brevifolia* (Pacific Yew Tree). The PAC’s mode of action [25] involves the binding to and preventing microtubule disassembly, thus causing mitotic arrest, and the induction of apoptosis. While PAC and cis-Pt display high antitumor potency and efficacy against all subtypes NSCLC [12], this chemotherapy suffers from a lack of selectivity, dose-limiting toxicity, drug resistance, and metastasis which have plateaued the clinical efficacy at about 10–14 months.

**Figure 1:**
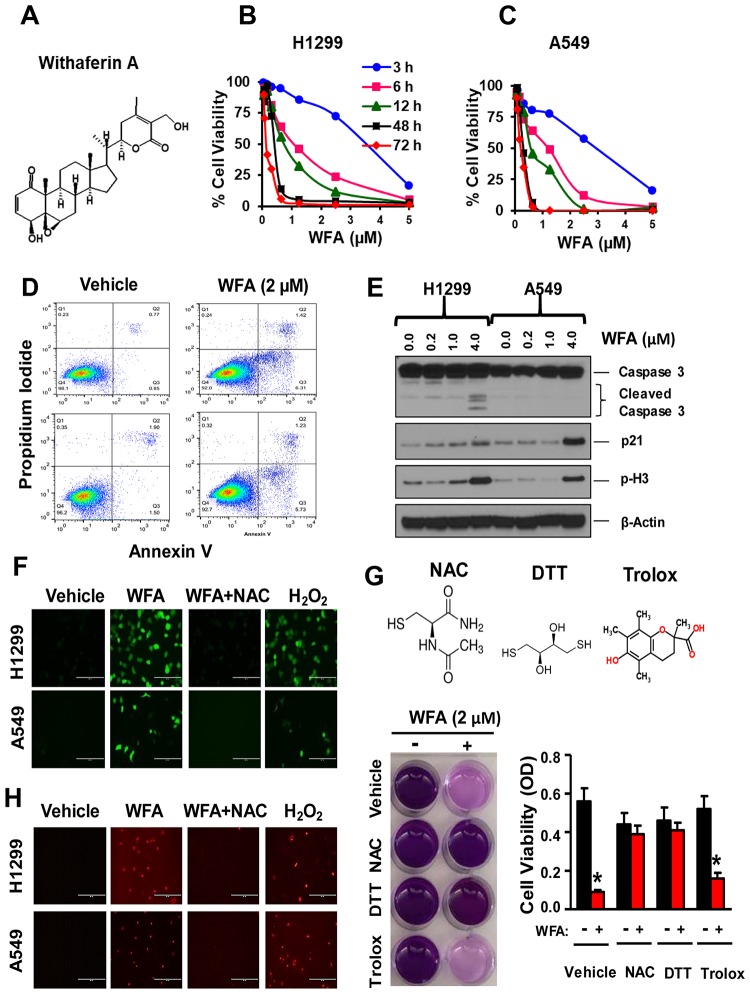
WFA inhibits NSCLC cell proliferation via thiol dependent induction of apoptosis. (**A**) Chemical structure of WFA. Cells were incubated with WFA for 3, 6, 12, 48 and 72 h and cell viability measured at 72 h. WFA dose-dependently inhibited the proliferation of H1299 (**B**) and A549 cells (**C**). (**D**) AnnexinV/PI assay depicting induction of apoptosis at 2 μM concentration of WFA compared to DMSO control. (**E**) Western blot analysis indicated increased expression of p21, phospho-H3, and cleavage of caspase-3 at different concentration of WFA (0, 0.2, 1, and 4 μM). ROS determination by fluorescent microscopy using the H2DCFDA assay (**F**) and Mitosox Red (**H**) indicated the induction of reactive oxygen species (ROS) production in H1299 and A549 cells. The induction of ROS by WFA was significantly inhibited in the presence of the thiol donor, N-acetyl cysteine (NAC). H_2_O_2_ (100 μM) was used as a positive control. The antiproliferative activity of WFA was inhibited in the presence of thiol donors; NAC (2.5 mM) and dithiothreitol (DTT) but not in the presence of trolox (**G**). Where indicated, data are presented as mean ± SD of 3 technical replicates. ^*^ *p* < 0.05.

In the present study, we demonstrate that withaferin A (WFA), a plant-derived steroidal-lactone anticancer compound significantly enhances the efficacy of PAC against human NSCLC cell lines. WFA (Figure 1A), a member of a large group of compounds collectively called withanolides was first isolated [26] from the alcoholic extracts of the Indian Ayurvedic medicinal herb, *Withania somnifera* (Ashwagandha). In the past decade, WFA has been widely investigated in preclinical studies [27] for its antitumor activity against lung [28–31], breast [32–34], uterine and cervix [35], ovarian [36], pancreatic [37], B-cell lymphoma [38]. Attractively, recently published studies [36, 39, 40] have demonstrated that sub-cytotoxic concentrations of WFA synergize the efficacy of standard chemotherapeutic drugs. Currently, our findings demonstrate that various combinations PAC and WFA are highly synergistic against the proliferation of the human NSCLC cells, H1299 and A549. Moreover, WFA was active against PAC-sensitive and PAC-resistant NSCLC cells thus demonstrating the potential therapeutic efficacy of WFA alone, and in combination with PAC against NSCLC cells and providing a strong rationale for further testing to advance this combination in clinical trials.

## RESULTS

### WFA inhibits the proliferation of NSCLC cells via thiol oxidation

To determine the antiproliferative effects of WFA (Figure 1A) on NSCLC cells, H1299 cells (large cell carcinoma) and A549 cells (adenocarcinoma) were seeded in 96-well plates (3000 cells/well) and incubated with WFA (0–5 μM) for 3–72 h. WFA (IC50: 0.20–0.68 μM) dose and time-dependently decreased the viability of both H1299 and A549 cells (Figure 1B, 1C). The highest inhibition of cell proliferation was observed at 48 h and 72 h in both cell lines. Concentrations of WFA ≤ 2 μM caused >90% inhibition of cell proliferation. Next, we examined whether WFA induced apoptosis in human NSCLC cells using AnnexinV/PI staining assay. WFA (2 μM) significantly increased in the percentage of annexin-V positive cells (Figure 1D). The induction of apoptosis was further confirmed by Western blot analysis (Figure 1E), depicting a dose-dependent increase in the cleavage of caspase-3, the expression of p21 and phospho-Histone 3 (p-H3).

Reactive oxygen species (ROS) generation has been shown to be critical for the anticancer activity of WFA against breast, ovarian and melanoma tumor cells [27, 41]. To investigate this hypothesis, H1299 and A549 cells were seeded in 6-well plates and incubated with 2 μM WFA for 12 h. ROS production was detected by fluorescence microscopy using H2DCFDA (Figure 1F) and Mitosox Red (Figure 1H) assays per manufacturer’s instructions. WFA (2 μM) increased the production of ROS in both H1299 and A549 cells (Figure 1F and 1H; Supplementary Figure 1). Interestingly, concomitant treatment of cells with N-acetyl cysteine (2.5 mM) inhibited the effects of WFA on ROS production. Moreover, the thiol-containing compounds NAC (2.5 mM) and dithiothreitol (DTT, 100 μM) completely abrogated the anticancer activity of WFA. Contrastingly, trolox, a non-thiol-containing ROS quencher did not inhibit the antiproliferative activities of WFA (Figure 1G). Thus, our results strongly suggest that the activity of WFA is highly mediated via thiol-dependent mechanisms.

### Synergistic effects of PAC, CisPt, and WFA on NSCLC cellular proliferation

**Figure 2:**
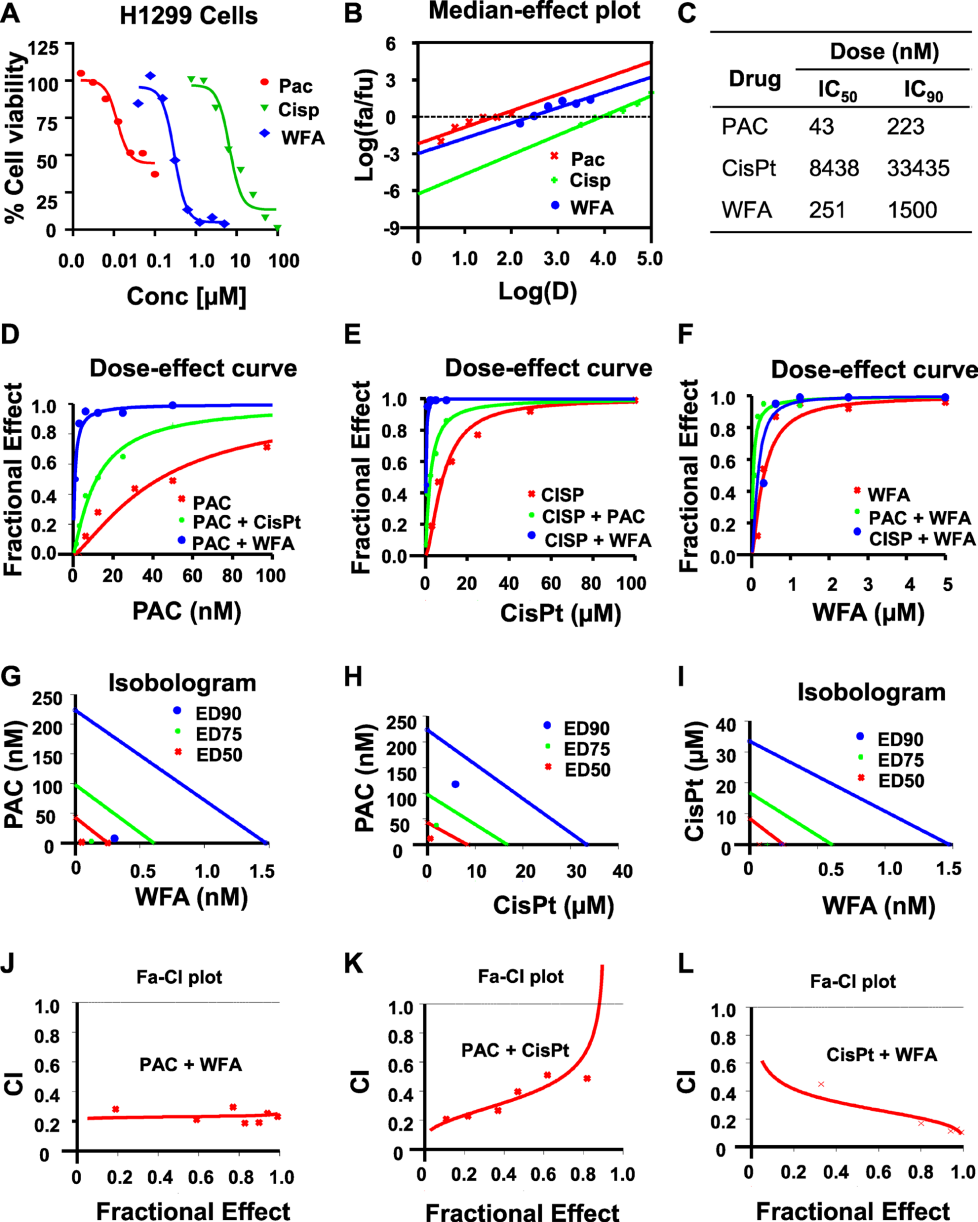
WFA synergizes the antiproliferative activity of PAC and CisPt against H1299 cells. The potency and efficacy of PAC, CisPt, and WFA against H1299 cells were compared using the median-effect equation. (**A**) Dose-response plots depicting PAC, CisPt and WFA dose-dependent inhibition of cell proliferation of H1299 cells. (**B**) Median-effect plot indicated PAC had lowest IC_50_ followed by WFA and CisPt the highest. Preliminary combinations of PAC and CisPt, PAC and WFA and CisPt and WFA were tested. PAC+WFA (**C**) displayed the greatest efficacy, followed by CisPt + WFA (**D**–**F**). Isobologram analysis (**G**–**I**) and combination index (CI) values (<1) indicated that all the combinations were highly synergistic against H1299 cells (**J**–**L**).

**Figure 3:**
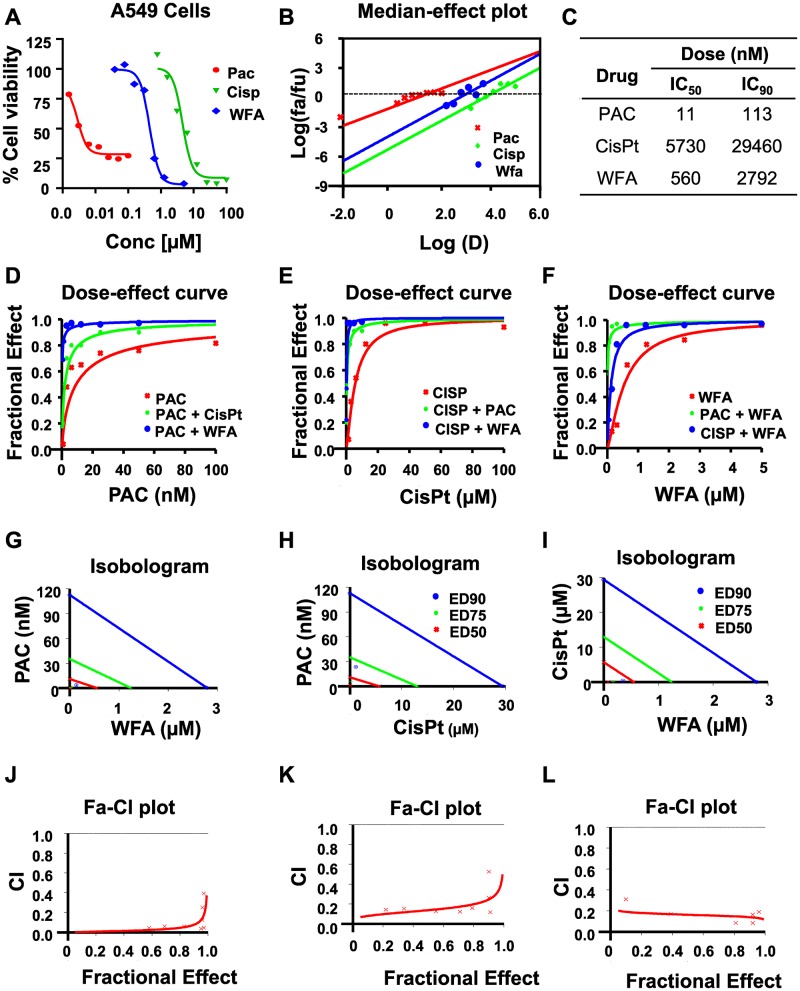
WFA synergizes the antiproliferative activity of PAC and CisPt against A549 cells. The potency and efficacy of PAC, CisPt, and WFA against A549 cells were compared using the median-effect equation. (**A**) Dose-response plots depicting PAC, CisPt and WFA dose-dependent inhibition of cell proliferation of A549 cells. (**B**) Median-effect plot indicated PAC had lowest IC_50_ followed by WFA and CisPt the highest. Preliminary combinations of PAC and CisPt, PAC and WFA and CisPt and WFA were tested. PAC+WFA (**C**) displayed the greatest efficacy, followed by CisPt + WFA (**D**–**F**). Isobologram analysis (**G–I**) and combination index (CI) values (<1) indicated that all the combinations were highly synergistic against A549 cells. Dose response curves were generated for WFA, PAC and combinations using Calcusyn 2.0 (Biosoft). A fractional effect of 1 means 100% cell kill by the drug (s), and zero means no effect (**J**–**L**).

In this study, we determined the potential synergistic effects of PAC, Cis-Pt, and WFA in H1299 and A549 cells *in vitro*. Firstly, we tested these compounds as individual agents and then based on our preliminary findings (Figures 2A and 3A), we tested the following combinatorial ratios: 1:40, 1:100, and 10:1 of PAC: WFA, PAC: cis-Pt, and cis-Pt: WFA, respectively. In H1299 cells, as individual drugs, PAC, Cis-Pt, and WFA all dose-dependently inhibited the cellular viability (Figure 2A). The median-effect plots (Figure 2B) show that PAC displayed the greatest potency (IC_50_: 43 nM), followed by WFA (IC_50_: 251 nM) and then Cis-Pt (IC_50_: 8438 nM) (Figure 2C). Next, we assessed whether the combinations of each drug with one of the other two compounds were synergistic against H1299 cells. Dose-response data (Figure 2D–2F) and isobologram analyses (Figure 2G–2I) show that the data points for; PAC+WFA, PAC+cis-Pt, and cis-Pt+WFA were all below the lines of additivity at IC_50_, IC_75_and IC_90_. Visual inspections of CI-Fa plots (Figure 2J–2L) indicate that the CI values at various effect levels were also <1. Therefore, the PAC+WFA, PAC+cis-Pt, and cis-Pt+WFA combinations were all synergistic against human NSCLC cells, H1299, and A549. To determine most synergistic combination, analysis of dose-reduction index (DRI) data indicated a 33-fold and 4-fold reduction in the IC_50_ of PAC against H1299 cells in the PAC+WFA and PAC+Cis-Pt combinations, respectively. For cis-Pt, we found a 26-fold and 7-fold change in IC_50_ when combined with WFA and PAC, respectively. Furthermore, the changes in WFA IC_50_ were 5-fold and 8-fold when combined with PAC and Cis-Pt, respectively.

Similarly, PAC, Cis-Pt, and WFA dose-dependently inhibited the proliferation of A549 cells (Figure 3) The median-effect plots (Figure 3B) showed that PAC had the lowest IC_50_ (11 nM), followed by WFA (IC_50_: 560 nM) and then Cis-Pt (IC_50_: 5730) (Figure 3C). As was observed for H1299 cells, all combinations were synergistic with PAC+WFA showing the strongest synergism (PAC DRI: 83-fold) followed by Cis-Pt+WFA (Cis-Pt DRI: 22-fold).Dose-effect curve (Figure 3D–3F, Isobologram analysis (Figure 3G–3I) and combination index (CI) values (<1) (Figure 3J–3L) indicated that all the combinations were highly synergistic against A549 cells.

### The effect of different ratios on the synergism of PAC and WFA against NSCLC cells

Since we found that PAC+WFA resulted in greater synergism, we further examined whether different combination ratios, cell numbers, sequences and schedules of treatment would alter the synergism. First, we tested PAC+WFA at 1:40, 1:20, and 1:10 ratios of PAC to WFA, respectively, on the viability of cells. At all the ratios tested, PAC+WFA were highly synergistic (CI<1) but the 1:40 ratio of PAC: WFA produced the strongest synergism in both cell lines (Tables 1 and 2). Next, we determined the most effective strategy for combining PAC and WFA by comparing the synergism obtained when the cells were exposed to both drugs: (1) concomitantly, (2) sequentially, and (3) in a schedule-dependent manner. While all the three treatment strategies resulted in synergism in PAC+WFA (Table 2), the synergistic effects were not scheduled dependent. However, pretreatment of cells with WFA (2 h) prior to incubation with PAC+WFA resulted in much greater synergism than pretreatment with PAC or simultaneous treatment. Also, in our preliminary studies shorter drug incubation durations (3 h) resulted in the much greater synergism of PAC+WFA than was observed when cells were exposed to drugs for extended drug periods (48, 72 h) (Supplementary Figure 2).

**Table 1:** Synergism summary of PAC and WFA on H1299 cells

Test agent	CI Values			Dm	m	r
	ED50	ED75	ED90			
PAC	N/A	N/A	N/A	25	0.6	0.99
WFA	N/A	N/A	N/A	190	1.1	0.98
PAC + WFA (1:40)	0.90	0.76	0.68	3.60	1.23	0.99
PAC + WFA (1:20)	0.89	0.82	0.83	6.22	1.04	1.00
PAC + WFA (1:10)	0.59	0.63	0.80	6.43	0.82	0.96

**Table 2:** Synergism summary of PAC and WFA on A549 cells

Test agent	CI Values			Dm	m	r
	ED50	ED75	ED90			
PAC	N/A	N/A	N/A	23	0.6	0.94
WFA	N/A	N/A	N/A	613	1.8	0.98
PAC + WFA (1:40)	0.80	0.66	0.68	7	1.5	0.94
PAC + WFA (1:20)	0.47	0.55	0.86	6	0.9	1.00
PAC + WFA (1:10)	0.55	0.52	0.71	9	0.9	0.99

**Figure 4:**
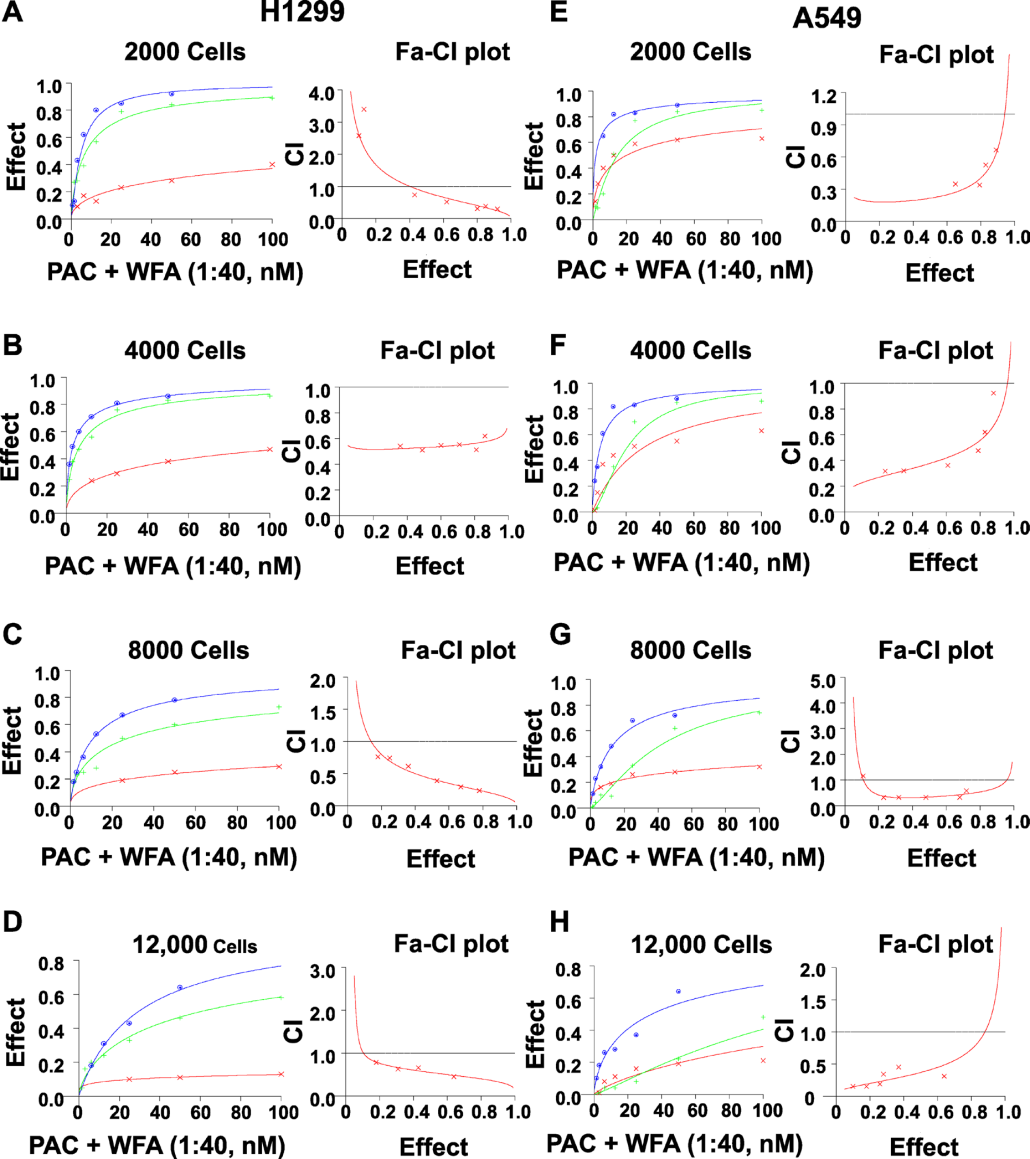
Increasing cell numbers did not alter the synergism of PAC and WFA against NSCLC cells. H1299 and A549 cells were plated at indicated densities and incubated with PAC and WFA alone, and in combination for 48 h. The efficacy and potency of PAC and WFA alone were greatly diminished with increasing cell number in both H1299 (**A–D**) and A549 cells (**E–H**). However, the combination of PAC and WFA synergistically inhibited the proliferation of both cell lines regardless of the number of cells plated. Cell viability was measured by MTT assay, fractional effects were calculated as per Chou *et al*. Synergism was assessed by the combination index (CI <1). Dose response curves were generated for WFA, PAC and combinations using Calcusyn 2.0 (Biosoft). A fractional effect of 1 means 100% cell kill by the drug (s), and zero means no effect.

Furthermore, we tested the effect of seeding increasing cell numbers (2000, 4000, 8000 and 12000 cells/well) on the synergism of PAC and WFA. For both H1299 and A549 cell lines, there was a dramatic decrease in the individual efficacy of PAC or WFA with an increase in the number of cells plated (Figure 4). As expected, the lowest IC_50_ values for PAC and WFA were observed when incubated with 2000 cells/well. However, when the cell numbers were increased to 12000 cells/well, there was up to 40-fold and 8-fold increase in the IC_50_ of PAC and WFA, respectively against both H1299 and A549 cells. Interestingly, whereas there was an increase in the combined IC_50_ values of PAC and WFA, there were no significant changes in the CI values at various cell numbers. Thus, we concluded that increasing the cell numbers of H1299 (Figure 4A–4D) and A549 (Figure 4E–4H) did not alter the synergism between PAC and WFA.

### Effect of PAC and WFA combination on colony formation and induction of apoptosis

**Figure 5:**
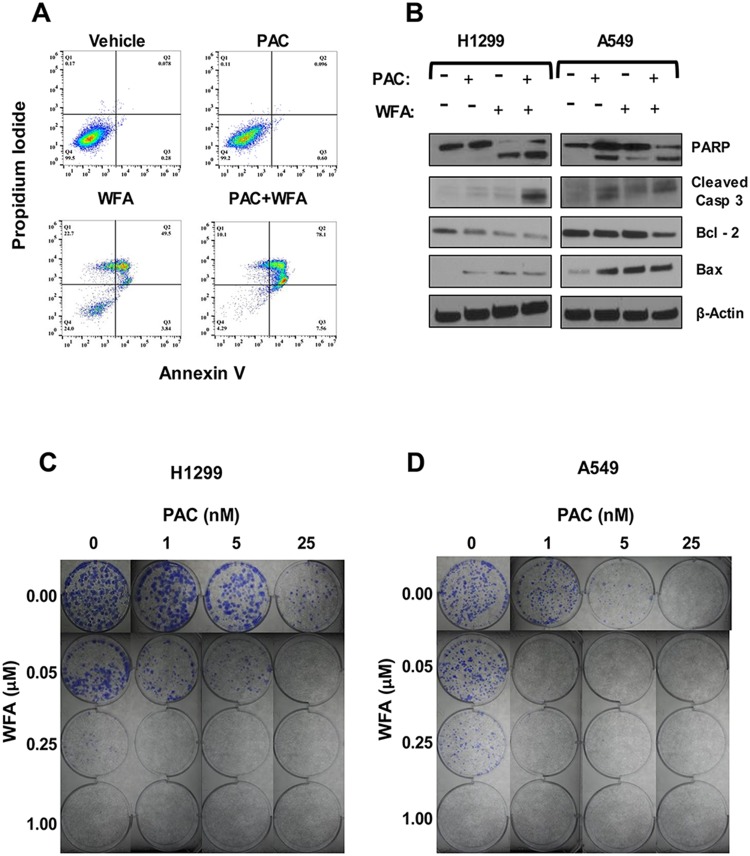
The combination of PAC and WFA synergistically induced apoptosis and inhibited colony formation in NSCLC cells. Apoptosis detection was performed by (**A**) Annexin-V/PI assay and (**B**) Western blot analysis. The combination of PAC and WFA synergistically increased the percentage of annexin-V positive cells as compared to each drug alone. There was an increase in the cleavage of PARP, caspase 3, increased Bax but decreased the expression of Bcl2. Representative images showing the colony formation assay in H1299 (**C**) and A549 (**D**) cells incubated with PAC and WFA alone and in combination. In both cell lines, PAC and WFA alone dose-dependently inhibited colony formation.

Further, we investigated whether the PAC + WFA combination induced apoptosis in NSCLC cells. Cells were incubated with WFA (1.0 μM) or PAC (25 nM), alone and in combination for 24 h, and apoptosis was detected by flow cytometry using the Annexin V/PI assay. We found that the combinations of PAC and WFA significantly increased the percentage of Annexin-V positive cells (Figure 5A). To support these findings, Western blot was performed, and the data obtained (Figure 5B) indicated an increase in the cleavage of both PARP and caspase-3, Bcl-2 degradation, and Bax upregulation. In addition, p21 and phospho-H3 were found to be significantly increased, an indication of cell cycle arrest and subsequent induction of apoptosis.

Next, to validate the MTT data, we investigated the potential effects of PAC and WFA combination on the replicative ability of H1299 and A549 cells using colony formation assay (Figure 5C and 5D). Briefly, cells (500 cells/well) were seeded in 6-well plates and incubated in media containing PAC (0–25 nM) and WFA (0–1 μM) alone, or their combination. PAC and WFA individually displayed dose-dependent inhibition of colony formation in H1299 (Figure 5C) and A549 (Figure 5D) cells. As expected, the combination of PAC and WFA inhibited colony formation in both cell lines greater than either agent used alone.

### Synergistic effect of PAC and WFA on migration and invasion of NSCLC cells

**Figure 6:**
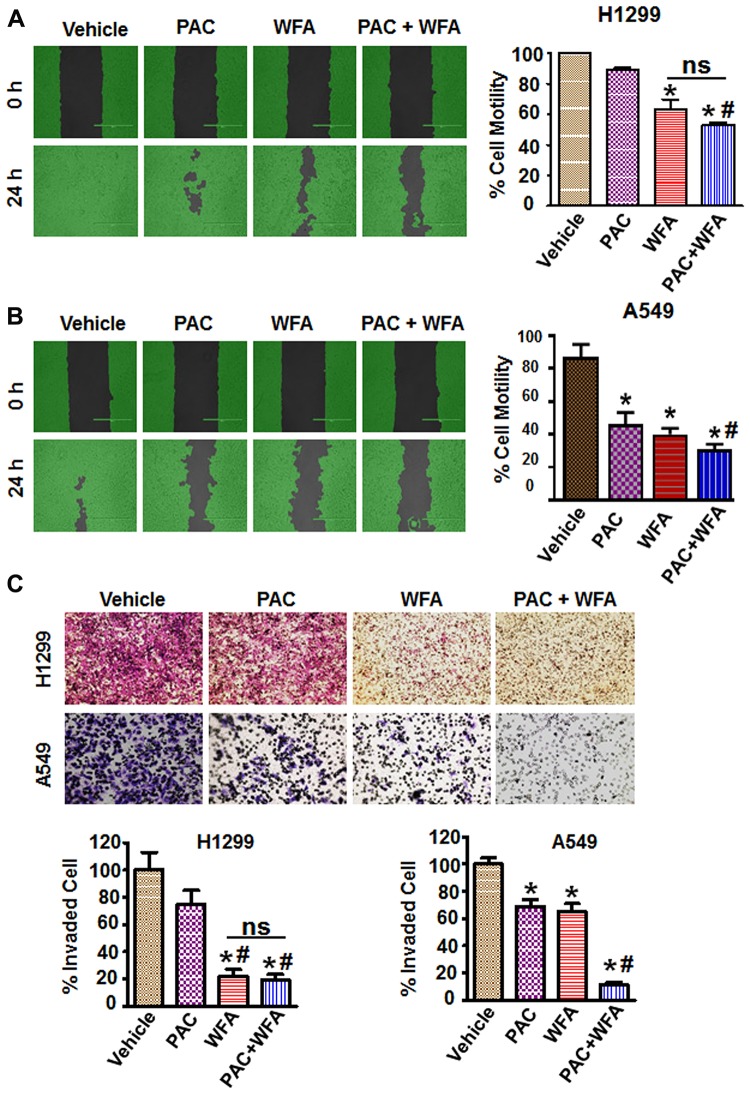
PAC and WFA synergistically inhibited NSCLC cell motility and migration. H1299 (**A**) and A549 (**B**) cells were incubated with PAC (5 nM) and WFA (0.2 μM) alone, and in combination. Percent cell motility was assessed by wound healing assay over 24 h and represented as mean ± SD. *p < 0.05. Student’s *t*-test was used to compare the treatments versus control. Pictures of wound areas were taken using a light microscope and analysis by Wimasis software. (**C**) Invasion assay depicting the synergistic inhibition of cellular trans-well migration in H1299 and A549 cells. A small blurred portion in the PAC alone (A549 cells) might have occurred due to a lack of proper focus and/or uneven staining. Data are mean ± SD and *p < 0.05. Significant difference between treatments and vehicle is shown by an asterisk whereas significance between PAC and/or WFA versus combination is shown by #. Data represent mean ± SD and *p < 0.05.

Cell motility, migration, and invasion are all important steps that are critical for the metastatic dissemination of NSCLC cells. Therefore, we investigated the effects of PAC and WFA, alone and in combination, on these cellular events. To determine effects on cell migration, we used the wound healing assay to assess the effects of PAC and WFA on cell motility into cell-free areas. Representative images (Figure 6) were obtained using a light microscope to monitor the wound areas between 0 and 24 h in order to determine the rate of cell motility. Low doses of PAC and WFA alone, inhibited the motility of H1299 (Figure 6A) and A549 (Figure 6B) cells. Comparatively, H1299 cells displayed greater migratory potential (>90% migration in 24 h) than A549 cells, thus the greatest effects on cellular migration for PAC and WFA were observed with A549 cells than H1299 cells. In both cell lines, at the tested concentrations, WFA displayed greater inhibitory effects on cell motility than PAC. However, in the presence of the combination of PAC and WFA, cellular motility was synergistically inhibited. (<10%, ^**^ *p* < 0.01). Furthermore, using the trans-well migration and invasion assays, we showed that PAC or WFA alone decreased migration and invasion compared to vehicle alone (Figure 6C).

### WFA targets PAC-induced chemoresistance in NSCLC cells

**Figure 7:**
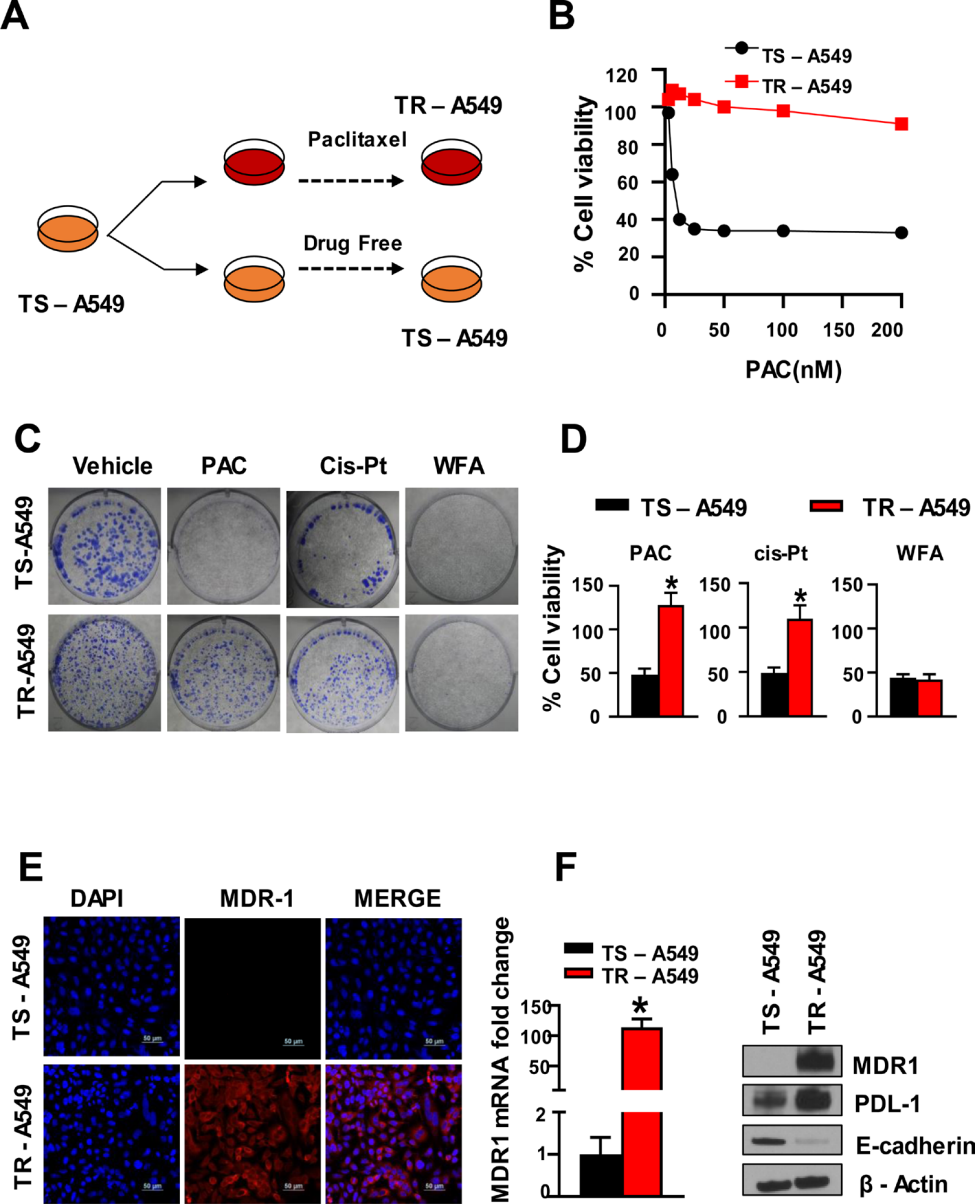
PAC chemoresistance in NSCLC cells. (**A**) Schematic representation of PAC-induced chemoresistance in A549 cells, (**B**) The cells incubated with PAC became resistant to PAC (TR-A549) while those incubated in media without PAC remained sensitive to PAC (TS-A549). (**C**) Colony formation assay and (**D**) MTT assay, indicated decreased sensitivity of TR A549 cells to both PAC and CisPt but the cells remained sensitive to WFA. Student’s *t* test was used to compare the effect of treatment versus control. (**E, F**) Confocal imaging, (F) RT-PCR and Western blot analysis indicated a significant increase in the expression of multi-resistant drug-protein (MDR1).

Previously published studies have reported that PAC treatment is associated with the induction and development of chemoresistance in NSCLC cells [42–44]. For example, Davi *et al.* [42] have shown that PAC-resistance was developed in human NSCLC cell lines by culturing these cells for >4 months in the presence of increasing concentrations of PAC or cis-Pt. In the present study, we used PAC-resistant cells (TR-A549 cells) that were developed as described previously (Figure 7A) [45] and kindly provided as a gift by Dr. Bruce Zetter (Harvard Medical School). Briefly, these TR-A549 cells were developed by starting PAC treatments 1/2 PAC IC_50_ concentrations for 2 d followed by culturing the surviving cells in drug-free media for 2 wks [46]. In subsequent cycles, the last PAC concentration of the previous cycle was increased 2-fold until the development of chemoresistance. The taxol-sensitive parental (TS-A549 cells) and taxol-resistant variants (TR-A549 cells) were characterized for their drug response to PAC until we observed a 10-fold change in IC_50_. In our studies, (Figure 7B) we evaluated the sensitivity of both the drug-sensitive and drug-resistant cells to PAC. Using the MTT and colony formation assays, we found that the PAC-resistant cells (TR-A549 cells) displayed cross-resistance between PAC and cis-Pt but remained sensitive to WFA (Figure 7C, 7D). Consistent with previous studies [42], we detected increased levels of mRNA and protein expression of MDR1 and PDL-1 in the drug-resistant NSCLC cells (Figure 7E, 7F).

**Figure 8:**
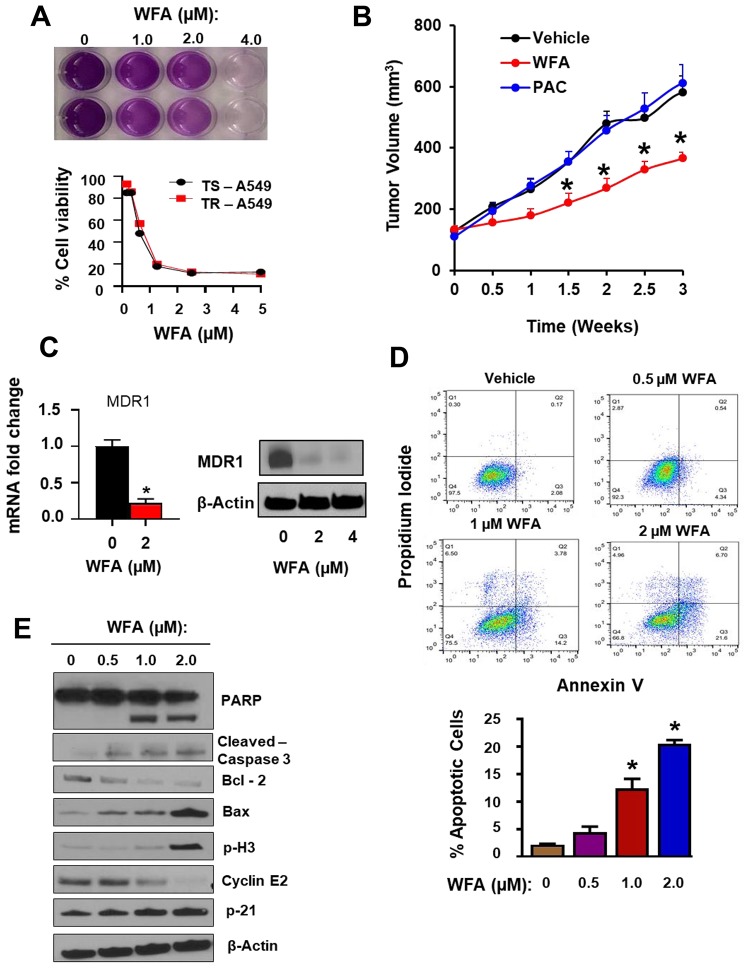
WFA alone inhibited the growth and proliferation of resistant PAC resistant NSCLC cells. (**A**) MTT assay depicting the *in vitro* inhibition of cellular proliferation of TR A549 and TS A549 (**B**) WFA significantly inhibited the growth of TR A549 (2.5 × 10^6^ cells/mouse) tumor xenografts in athymic nude mice. (**C**) RT PCR and Western blot analysis indicated dose-dependent inhibition of the expression MDR1 in TR A549 after incubation with WFA for 48 h. (**D**) Annexin V/PI assay indicated dose-dependent induction of apoptosis in TR A549 cells after incubation with WFA (0–2 μM) for 24 h. (**E**) Western blot analysis confirmed the induction of apoptosis as indicated by the increased cleavage of PARP and caspase 3, increased expression of Bax, p21, phosphor-H3 while inhibiting the expression of Bcl2 and cyclin E2.

To evaluate the response of PAC-resistant TR-A549 cells to WFA, we explored the antitumor effects of WFA on the growth and proliferation of TR-A549 cells both *in vitro* and *in vivo* (Figure 8). First, using *in vitro* assays, we demonstrated that both the parental cells (TS A549) and drug-resistant variants (TR-A549) remained sensitive to WFA *in vitro* (Figure 8A). To determine the effects of WFA on PAC-resistant cells *in vivo*, we established subcutaneous xenografts of PAC resistant cells in mice. WFA (10 mg/kg) was administered intraperitoneally in 3 doses per week in athymic nude mice xenografted with 2 × 10^6^ TR-A549 cells/mouse (Figure 8B). In agreement with *in vitro* data, mice treated with PAC (10 mg/kg divided into 3 doses a week) did not show any difference in tumor volume compared to those treated with vehicle. However, WFA significantly and time-dependently decreased the average tumor volumes of TRA549 xenografts when compared to vehicle and PAC groups. Mechanistically, WFA inhibited the mRNA and protein expression of MDR1 in TR A549 cells (Figure 8C); however, modulation of PD-L1 with WFA remains to be determined. AnnexinV/PI assay indicated dose-dependent induction of apoptosis in TR A549 cells after incubation with WFA (0–2 μM) for 24 h. The induction of apoptosis was significant at the concentrations of 1 μM WFA or higher (Figure 8D). Western blot analysis confirmed the induction of apoptosis as indicated by the increased cleavage of PARP and caspase 3, increased expression of Bax, p21, phosphor-H3 while inhibiting the expression of Bcl2 and cyclin E2 (Figure 8E). Therefore, our data presented here show that in addition to synergizing the effects of PAC, WFA targets both drug-sensitive and drug-resistant NSCLC cells. As such, the ability of WFA to synergize the anticancer efficacy of PAC and cis-Pt in drug-sensitive NSCLC cells, and target both drug-sensitive and drug-resistant NSCLC cells provides a strong rationale for the development of a combination of PAC and WFA for the treatment of NSCLC.

## DISCUSSION

NSCLC is a highly heterogeneous group of tumors characterized by multiple genetic alterations resulting in several dysregulated cellular signaling pathways. As such, these tumors are biologically very aggressive and rarely curable with current treatment strategies. Today, taxane-platinum chemotherapeutic combinations represent the standard of care as first-line regimens for all sub-types of advanced NSCLC with no targetable genetic mutations [8, 10, 21]. PAC, a member of the taxanes, is a major component of taxane-platinum systemic therapy for NSCLC [8, 10, 21]. Although efficacious, the clinical response rate is only 20–30% and the maximum survival efficacy has plateaued at about 14 months [8, 9]. Dose-limiting toxicity, chemoresistance, and metastasis are major clinical obstacles to PAC treatment for advanced NSCLC [6]. Therefore, there is a significant unmet clinical need to develop safe and efficacious alternative therapies for advanced NSCLC. In the present study, we evaluated the therapeutic potential of a combination of WFA and PAC on NSCLC by investigating the antitumor effects of WFA alone or alongside PAC against human NSCLC cell lines. Our findings demonstrate that various combinatorial ratios of WFA and PAC were highly synergistic against the growth, proliferation, migration and invasion of the human NSCLC cells, H1299 and A549.

Previously, multiple studies have extensively examined and demonstrated the anticancer effects of WFA on various cancer types [27]. More attractively, it has been shown that in addition to its cytotoxicity, WFA has a synergistic effect on several standard chemotherapeutic drugs against tumor cell proliferation [36, 39, 40]. In line with these research hypotheses, we aimed to determine the synergistic effects of WFA alongside PAC and cis-Pt against human NSCLC cells. This combination strategy is particularly exciting for the case of NSCLC since the clinical efficacy with the current platinum-taxane combinations seems to have reached a maximum [36]. In agreement with published studies [23, 24], our data show that PAC displays greater potency (lowest IC_50_) against both H1299 and A549 human NSCLC cell lines compared to cis-Pt. This observation provided us with the basis to consider PAC as the standard drug and we determined whether its combination with WFA would result in much greater efficacy than either drug used alone. In our experimental approach, to determine whether the interaction was more than additive, synergism analysis was performed using the methods developed by Chou *et al.* [47]. Several studies have used CI and DRI to investigate the synergistic effects of anticancer drugs against breast [32], lung, pancreatic [40] and ovarian [39] cancers. Similarly, in our studies, we demonstrated that WFA dramatically increased the efficacy and potency of both PAC and cis-Pt in a synergistic manner (CI<1). Interestingly, the changes in IC_50_ values were much dramatic for PAC (>40-fold) than were observed for cis-Pt or WFA. Thus, we concluded that WFA alongside PAC resulted in much greater synergism than any other combination tested. Also, as was reported by Liu *et al*., we investigated whether the synergism of PAC and WFA could be enhanced by altering the sequence, concentration, schedule and duration of drug exposure [48–50]. Our findings show that the synergistic effects of PAC and WFA were not scheduled dependent but preincubation of H1299 and A549 cells with WFA dramatically enhanced the efficacy of the combination. Thus, as was reported previously [27], WFA had a chemo-sensitizing effect on PAC against NSCLC cells. The mechanisms by which WFA increased the sensitivity of NSCLC cells to PAC were not explored in the present study.

Tumor cell chemoresistance to taxane-platinum chemotherapy is a major therapeutic challenge in the management of NSCLC. In fact, several studies [21, 42, 46] have shown that chronic PAC/cis-Pt treatment induces tumor cell mechanisms that promote chemoresistance and metastasis in NSCLC cells. This prompted us to determine whether WFA was active against PAC-resistant NSCLC cells (TR-A549). In these studies, we used a PAC-resistant (TR-A549) cell line that expressed high levels of drug efflux protein, MDR1, indicating that increased drug-efflux mediated chemoresistance to PAC [21]. In addition, these cells also expressed high levels of PDL-1, which strongly suggests a link between the simultaneous development of drug resistance and immune evasion in NSCLC [51]. Since we found that WFA was active against this MDR-1 overexpressing TR-A549 cell line, it appears that WFA is not a substrate for MDR-1. In addition, the TR-A549 cells displayed decreased E-cadherin expression, indicating an EMT phenotype which strongly suggests that EMT also plays a critical role in chemoresistance in NSCLC [43, 52]. In a recent study [53], we have shown that WFA inhibits EMT in NSCLC cells, and we therefore strongly hypothesize that the use of PAC and WFA can prevent the emergence of drug resistance and metastasis in NSCLC. Together, these novel findings strongly support our rationale for combining PAC with WFA, and further suggest that this strategy has an additional benefit of the ability to prevent the emergence of drug resistance and metastasis in NSCLC.

In conclusion, our findings have several potential clinical implications for the treatment of advanced NSCLC. Firstly, we have shown that WFA alone is highly active against the growth and proliferation of two human NSCLC cell lines, H1299, and A549. Secondly, our data demonstrate that the combinations of PAC and WFA, at various combination ratios were highly synergistic against both H1299 and A549 cells *in vitro*. Since cis-Pt alongside PAC is the current first-line chemotherapy for NSCLC without driver genetic mutations, we have shown that PAC with WFA is more potent and more efficacious than the standard of care. Therefore, PAC+WFA represents a viable and attractive alternative therapeutic strategy to platinum-based chemotherapy for the treatment of advanced NSCLC with no targetable driver mutations. More importantly, WFA was active against both drug-sensitive (TS-A549) and drug-resistant (TR-A549) cells which further broadens the scope and relevance of the PAC+WFA combination in NSCLC. Together, these findings provide a strong rationale for the development of PAC and WFA as a therapeutic alternative to platinum-based therapies.

## MATERIALS AND METHODS

### Drugs, chemical supplies, and reagents

Withaferin A (purity, > 95%) was provided as a gift sample by 3P Biotechnologies (Louisville, KY, USA), paclitaxel (Cat # P-9600) was purchased from LC laboratories (Woburn, MA, USA), cisplatin (cis-diamine platinum [II] dichloride, Cat # P4394) and 2.3% crystal violet solution (Cat # HT90132) were purchased from Sigma Aldrich (St. Louis, MO, USA). MTT (3-[4,5-dimethylthiazol-2-yl]-2,5-diphenyltetrazolium bromide, Cat #L11939) was purchased from Alfa Aesar (Ward Hill, MA, USA) while dimethyl sulfoxide (DMSO, Cat #13390) was purchased from Electron Microscopy Sciences (Hatfield, PA, USA). Dulbecco’s Modified Eagle’s Medium (DMEM, Cat #10569044), RPMI medium 1640 (Cat # 11875-095), 0.25% Trypsin-EDTA (Cat # 25200-072), antibiotics (100 U penicillin/100 mg streptomycin, Cat# 15140), and ultrapure distilled water (Cat #10977-015) were purchased from Life Technologies (Grand Island, NY, USA). Heat-inactivated fetal bovine serum (FBS, Cat# 12306C)) was purchased from SAFC (St. Louis MO, USA). Pierce RIPA cell lysis buffer (Cat #89901), 100× halt protease/phosphatase inhibitor cocktail (Cat #7844), Pierce BCA protein assay kit (Cat #23225), PVDF-transfer membrane (Cat#88518), Spectra Multicolor broad range protein ladder (Cat #26634) and Pierce ECL Western blotting Substrate (Cat # 32106) were purchased from ThermoScientific (Rockford, IL, USA). Bolt 4-12% Bis-Tris phosphate gels (Cat #NW04125Box, NW04120Box, NW04122Box) and 20× Bolt MES running buffer (Cat# B002) were purchased from Life Technologies (Carlsbad, CA, USA). The FITC-AnnexinV/PI Apoptosis Assay kit (Cat#V13242) was purchased from Life Technologies (Eugene, OR, USA). Primary monoclonal antibodies (against β-actin, PARP, P21, Bcl2, Bax, phospho-histone3, phospho-cdc2, cyclin E_2_, cyclin B_1_, cyclin A1, cleaved –caspase 3, cleaved-caspase-7, cleaved-PARP) as well as secondary antibodies (anti-mouse IgG and anti-rabbit IgG) were purchased from Cell Signaling Technology, Inc. (Danvers, MA, USA).

### Cell lines and culture conditions

The human NSCLC cell lines, H1299, and A549 cells were obtained from American Type Culture Collection (Manasa, VA, USA). These cell lines were maintained in either DMEM or RPMI 1640 culture media (10% FBS, 1% antibiotics) for A549 and H1299 cells, respectively. The cultures were incubated at 37°C in a 5% CO_2_ humidified incubator and passaged at ~80% confluence for less than 20 cycles.

### Drugs and treatments

Stock solutions of PAC, WFA, and CisPt at concentrations of 5 mM were prepared by dissolving in 100% DMSO at 25 °C. The stock solutions for each drug were stored in aliquots of 40 μL at -20°C until used in subsequent experiments. Cells were incubated with the drugs after diluting the stock concentrations of each test agent in cell culture media to desired concentrations. In all treatments, DMSO was kept at a maximum of 1% in the vehicle and test treated groups.

### MTT cell viability assay

Cell viability was measured indirectly using the MTT colorimetric assay. Briefly, A549 or H1299 cells were seeded in 96-well plates and incubated at 37° C overnight to allow attachment. Following cellular attachment, the cell culture media was discarded and replaced with fresh cell culture media containing different concentrations of drugs/agents. These cells were incubated with the drugs for designated periods and then incubated with cell culture media containing 0.5 mg/mL MTT reagent solution. After 3 hours, the MTT containing media was discarded and the purple formazan crystals in each well were solubilized using 200 μL of DMSO. The absorbance of the resulting solution was determined spectrophotometrically by measuring the optical density (OD) at 570 nm. The OD values of the solution in the wells containing untreated cells were considered as 100% cell growth and used as a reference to calculate the percent growth of other wells. The arithmetic mean of 3 technical replicates was calculated at each concentration of PAC and WFA alone and in combination to obtain the percent cell viability. Data were expressed as mean±SD of more than 3 separate experiments.

### Colony formation assay

To perform the colony formation assay, H1299 and A549 cells were seeded into 6-well tissue culture plates at a density of 500 cells/well in 2 mL of medium (10% FBS) and incubated in a 37 °C humidified atmosphere containing 5% CO_2_ for 24 h. These cells were then incubated in culture medium containing different concentrations of either PAC or WFA, alone and in combination for 24 h. The drug-containing medium was discarded and replaced with fresh drug-free medium. After 7 d, the plates were washed with sterile PBS, and the cells were fixed using methanol/acetic solution (3:1) for 5 mins and stained with 0.5% crystal violet (in methanol) for 15 min. The crystal violet solution was carefully removed, the plates were rinsed in a stream of running water and left to air dry at room temperature. The number of colonies in each well was counted under a microscope.

### Apoptosis analysis by flow cytometry

Apoptosis was detected using the Annexin V/propidium iodide (PI) assay. Briefly, H1299 and A549 cells were treated with PAC and WFA alone and in combination for 24 h. Both floating and attached cells were collected, washed twice with ice-cold PBS and re-suspended in 100 μL of Annexin-binding buffer at a cell density of approximately 1 × 10^6^ cells/mL. To each 100 μL of cell suspension, 5 μL of FITC-Annexin V and 1 μL of 100 μg/mL PI solution was added and incubated for 15 minutes in the dark. After incubation, 400 μL of Annexin binding buffer was added to each Annexin V/PI stained cell suspension and analyzed for fluorescence using a flow cytometer by measuring the fluorescence emission at 530 nm and 575 nm. A total of 10,000 cells were counted in each cell suspension, and the data were analyzed using FlowJo software (Trista, CA, USA) to obtain the percent early and late apoptotic cells.

### Cell migration and motility assays

The wound healing assay was performed to assess cellular migration and motility. This was accomplished using the 2-well culture inserts (ibidi^®^) following the manufacturer’s instructions. Briefly, ibidi culture inserts were placed in 6-well plates and 3 × 10^5^ cells/ml were seeded into each of the two chambers of the same insert. The cells were incubated in the inserts for 24-h to obtain confluent monolayers and the inserts were removed to create a wound area (gap) between the two cell growth areas. Floating and dead cells were removed by washing the cells twice using culture media, then media containing PAC or WFA alone and in combination was added. Cellular migration and motility were assessed using a bright-field microscope at 0 and 24 h. The migrated cells were photographed using a microphotographic camera, and the cell-covered areas were measured using WimScratch software program (Wimasis, Munich, Germany).

### Transwell migration and invasion assay

Cell migration and invasion was performed using transwell culture inserts pre-coated with or without 40 μL of 3.0 mg/mL Matrigel (BD). H1299 cells (4 × 10^4^) suspended in 200 μl of the serum-free medium in the presence and absence of test agent (s) were seeded onto the upper compartment of the transwell chamber. The lower chamber was filled with 600 μL of media supplemented with 10% FBS as an attractant to cause cell migration. After 24 h incubation, cells on the upper surface of the transwell insert were removed using a cotton swab. The migrated cells at the bottom of the insert were fixed using 4% paraformaldehyde, permeabilized using 100% methanol and stained using 0.2% toluidine blue (in 1% sodium borate). The number of migrated cells were counted under the light microscope.

### Western blot analysis

Whole cell protein lysates were prepared using RIPA buffer as per the manufacturer’s instructions. The total protein concentration was determined using BCA assay and an aliquot of 20 μg total cellular protein of each sample was resolved by SDS-PAGE on 4-12% Bis-Tris gels. The separated proteins were transferred to PVDF membranes, and these membranes were blocked using 5% non-fat dry milk in TBST for 1 h. To determine the relative expression of specific proteins, membranes were first probed with primary antibodies followed by the respective HRP-conjugated secondary antibodies. The expression levels of each protein were determined by visualizing the protein bands on the blots using the chemiluminescent Pierce ECL Western blotting. Relative band density for each protein was quantified using ImageJ software (NIH, Bethesda, MD, USA) and normalized to β-actin as a total protein loading control.

### Tumor xenograft studies in nude mice

Taxol resistant NSCLC cells (TR A549 cells) were used to determine the *in vivo* efficacy of WFA. Female athymic nude (nu/nu) mice (5–6-week old) were purchased from Charles River Labs and maintained in accordance with the Institutional Animal Care and Use Committee guidelines. TR A549 cells (2.5 × 10^6^ cells/mouse) in 100 μL of serum-free media were mixed (50:50) with Matrigel matrix (Becton Dickinson, Bedford, MA) and subcutaneously injected into the left flank of each mouse. The mice were provided purified AIN-93M diet and water ad libitum. Once average tumor size reached about 80–120 mm^3^, mice were randomly divided into 3 groups (*n* = 5): (i) treated with vehicle, (ii) PAC (10 mg/kg divided in 3 doses per week) and (iii) WFA (10 mg/kg) as 3 doses per week. All treatments for vehicle and intervention drugs were done via intraperitoneal injections (i. p) and the tumor volumes were measured twice weekly.

### Statistical analysis

Statistical analysis was performed using Graph Pad Prism 8.0 (La Jola, CA) and CalcSyn 2.0 (Biosoft, Cambridge, UK). Data are presented as means ± SD of at least 3 separate experiments. For cell proliferation assay, IC50 values were calculated. Data represented in the xenograft studies is the average ± standard error of 8-10 animals. Differences between the means of the treatments were calculated for tumor volume and *p*-values were determined by Student’s *t*-test. Dose response curves were generated for WFA, PAC and combinations using Calcusyn 2.0 (Biosoft). A fractional effect of 1 means 100% cell kill by the drug (s), and zero means no effect. To determine the synergistic interaction between PAC and WFA, the dose-effect data on percent cell viability was analyzed by the combination index (CI) method described by Chou *et al*. [47]. The CI values; CI<1, C=1 and C>1 indicated synergism, additive and antagonism, respectively. Differences were considered a priori to be statistically significant if the p value was less than 0.05.

## SUPPLEMENTARY MATERIALS


