# Supplementary material for: Synergistic combinations of paclitaxel and withaferin A against human non-small cell lung cancer cells
Source: Oncotarget. 2020 Apr 21;11(16):1399–416. doi: 10.18632/oncotarget.27519 (PMC7185067; doi:10.18632/oncotarget.27519)
Supplement: Supplementary file 1 [file oncotarget-11-1399-s001.pdf]

Synergistic combinations of paclitaxel and withaferin A against human non-small cell lung cancer cells

SUPPLEMENTARY MATERIALS

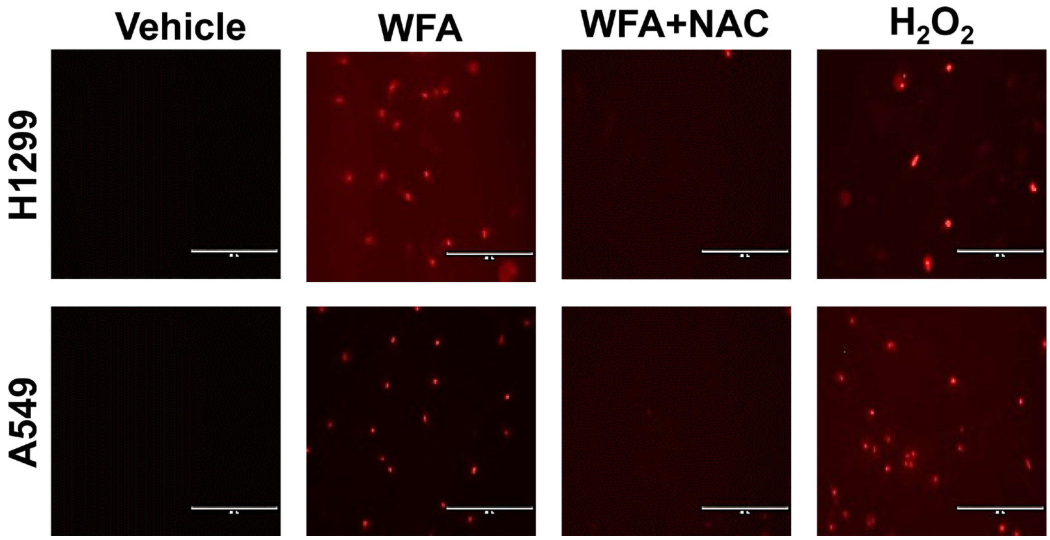

Supplementary Figure 1: ROS determination by fluorescent microscopy using MitoSox red. The ROS generation was assessed by confocal microscopy after staining with MitoSOX Red. Cells were seeded in chambered slide, and treated with Me2SO for 4 h. The cells were then exposed to MitoSOX Red (Invirtogen) for 15 min and 100 nM MitoTracker for 20 min at 37°C, fixed in 2% paraformaldehyde for 1 h at room temperature, washed, and the coverslips were mounted onto slides. Cells were observed under the fluorescent microscope.

Schedule dependent interaction of PAC and WFA against NSCLC cells

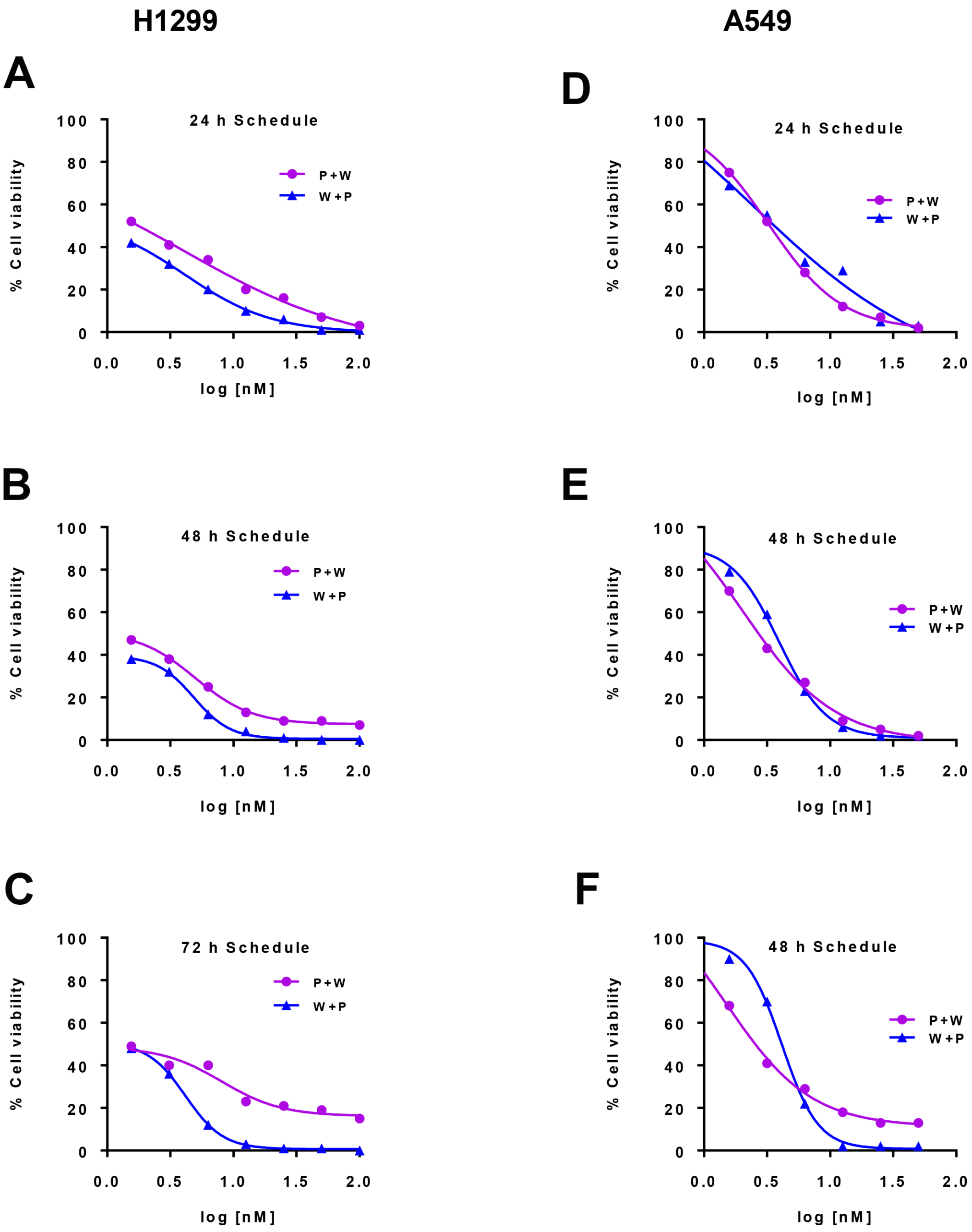

Supplementary Figure 2: Schedule dependent interaction of PAC and WFA against NSCLC cells. NSCLC cells were plated in the 96 well plate and treated first with paclitaxel followed by WFA or vice versa. After 24, 48 and 72 hr of incubation, cell growth was measured by MTT assay.
